# Spermitin: A Novel Mitochondrial Protein in *Drosophila* Spermatids

**DOI:** 10.1371/journal.pone.0108802

**Published:** 2014-09-29

**Authors:** Jieyan V. Chen, Timothy L. Megraw

**Affiliations:** Department of Biomedical Sciences, Florida State University, Tallahassee, Florida, United States of America; University of California Los Angeles, United States of America

## Abstract

Mitochondria, important energy centers in the cell, also control sperm cell morphogenesis. *Drosophila* spermatids have a remarkably large mitochondrial formation called the nebenkern. Immediately following meiosis during sperm development, the mitochondria in the spermatid fuse together into two large aggregates which then wrap around one another to produce the spherical nebenkern: a giant mitochondrion about 6 micrometers in diameter. The fused mitochondria play an important role in sperm tail elongation by providing a structural platform to support the elongation of sperm cells. We have identified a novel testis-specific protein, Spermitin (Sprn), a protein with a Pleckstrin homology-like (PH) domain related to Ran-binding protein 1 at its C-terminus. Fluorescence microscopy showed that Sprn localizes at mitochondria in transfected Kc167 cells, and in the nebenkern throughout spermatid morphogenesis. The role of Sprn is unclear, as *sprn* mutant males are fertile, and have sperm tail length comparable to the wild-type.

## Introduction

During *Drosophila melanogaster* spermatogenesis spermatids undergo synchronous differentiation in which many changes take place at the subcellular level, including remodeling of existing organelles (mitochondria, nuclei), formation of new organelles (flagella, acrosomes), polarization of elongating cell clusters (cysts), and plasma membrane addition [1]. As spermatids form immediately following meiosis II, cytoplasmic mitochondria fuse together into two giant aggregates, a process mediated by a testis-specific mitofusin called Fuzzy onions (Fzo) [2], [3]. The two aggregates then wrap around one another to produce the giant spherical nebenkern [2], [4]. During spermatid elongation, the nebenkern also elongates and it is one of the four major structures that contribute to the formation of the elongating sperm tails, along with actin bundles, cytoplasmic microtubules and the axoneme [5]. Mitochondrion proteins are required for nebenkern morphogenesis during spermatid elongation, such as proteins involved in mitochondrial fusion like Fzo, Rhomboid-7 and optic atrophy1 (Opa1), mitochondrial fission like Drp1, and other proteins involved in mitochondrial integrity including Pink1, Parkin and Dj-1 [3], [6]–[11]. In this study we report a novel testis-specific protein, Spermitin (CG14128, Sprn), which localizes to mitochondria and to the lumen of the nebenkern during spermiogenesis.

## Materials and Methods

### Plasmids and fly stocks

p*UAS-sprn-GFP* and p*Actin5C-sprn-Myc* were generated by cloning *CG14128* cDNA (IP13164, *Drosophila* Genomics Resource Center) through Gateway cloning into the pTWG-attB and pAWM vectors (Terence Murphy, The *Drosophila* Gateway Vector Collection, Carnegie Institution of Washington, Baltimore, MD) respectively. pTWG-attB was constructed by cloning a 368 bp fragment containing *attB* sequence from pVALIUM1 (2567–2935), generated by PCR, into the *Aat II* restriction site (at 1989 bp) of pTWG. *Sprn* mutant stock *CG14128^MB12149^* (referred to here as *sprn^MB12149^*) was obtained from the Bloomington *Drosophila* Stock Center. The stock was isogenized to *w^1118^* by backcrossing 6 generations. p*UAS-sprn-GFP* transgenic flies were made by GenetiVision Inc. (Houston, TX) via PhiC31-mediated chromosome integration on the third chromosome with VK20:(3R)99F8 as the docking site. The p*UAS-sprn-GFP* insertion was lethal and balanced with TM6B, Hu^1^ Tb^1^. GAL4 stocks were obtained from the Bloomington Drosophila Stock Center. Sprn-GFP was expressed ubiquitously using tubp-GAL4^LL7^ (stock #5138) [12], Act5C-GAL4^E1^ (stock #25374, FBrf0205896) [13] or Ubi-GAL4 (stock #32551, FBrf0212198) [14], and in larval brains using *elav-GAL4^C155^* driver (stock #458) [15]. For the lethal phase analysis, GAL4 ‘driver’ stocks were crossed to the p*UAS-sprn-GFP* stock. The appearance of non-Tubby (Tb^1^) larvae or pupae was evidence of survival from ectopic expression of Sprn-GFP except for the Act5C-GAL4^E1^ driver, which is balanced over SM5. In that case, we could only score survival to adulthood.

*attB* primers:

forward: AATTGACGTCCGCTGCATCCAACGCGTTGGGA

reverse: AATTGACGTCGAATTAGGCCTTCTAGTGGAT

### RT-PCR

**Figure 1:**
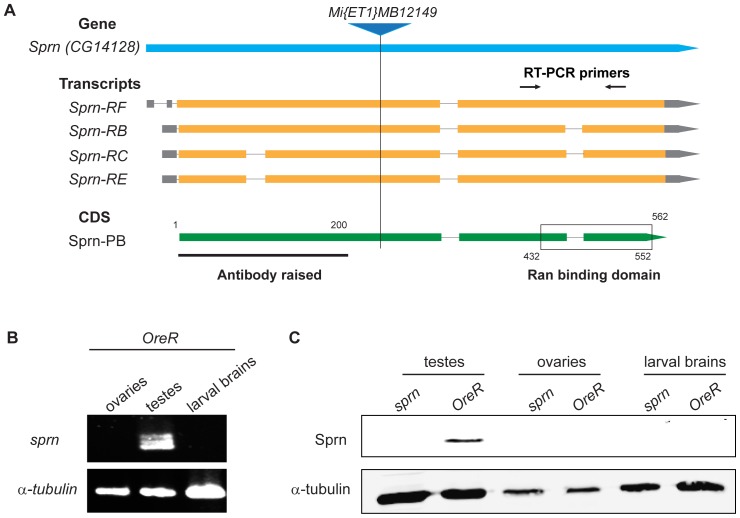
Sprn is expressed in testes. (A) Schematic diagram of the *sprn* gene, transcripts, and protein products showing the site of transposon insertion, the region the antibody was raised against, the conserved PH/Ran protein binding homology domain and the primers used for RT-PCR. For the transcripts, the coding exons are orange, the UTRs grey, and the introns depicted as thin lines. (B) Expression of *sprn* mRNA shown by RT-PCR from adult ovaries, testes, and third instar larval brains. Three distinct bands of approximately 500 bp were detected in testes but not in ovaries or larval brains. Two of the bands are likely the predicted 483 bp and 534 bp products from *sprn-RB/RC* and *sprn-RE/RF*, respectively. The third band is not readily accounted for, but could be a new alternative splice product. (C) Western blot of extracts from wild-type *OreR* or homozygous *sprn^MB12149^* mutant adult testes, ovaries, and third instar larval brains. Sprn in testes but not in ovaries or larval brains, anti-Sprn antibody recognizes a band around 68 kD consistent with predicted size, and Sprn is undetectable in the *sprn^MB12149^* mutant.

Larval brains, adult ovaries and testes were dissected in Dulbecco's Phosphate Buffered Saline (DPBS, Invitrogen) and RNA was isolated using TRIzol Reagent (Ambion). cDNAs were then generated using iScript Advanced cDNA Synthesis Kit (Bio-Rad). For *sprn* RT-PCR reactions, primers flanking intron 3 of *sprn-RB* (see Fig. 1A) were used; for *α-tubulin*, primers flanking intron 1 of *α-tubulin-RA* were used.

*sprn* primers:

forward: CCCATACCCTACCGACAAGATGCTGG

reverse: GGGTTCACGGAAGGAGCAGGCGATCC

*α-tubulin* primers:

forward: CGTATACGCTCTCTGAGTCAGACCTC

reverse: GCAGACCGGTGCACTGATCGGCCAGC

### Production of antibodies

DNA sequence encoding amino acids 1–200 of Sprn-PB were cloned into the pET100/DTOPO vector (Invitrogen) for expression of the 6XHis-tagged Sprn protein fragment in *E. coli* strain BL21(DE3)pLysE. The 6XHis-tagged protein was then purified by Ni^2+^-Immobilized metal affinity chromatography and used to immunize rabbits (Cocalico Biologicals, Reamstown, PA). For immunostaining, the rabbit serum was affinity-purified against the 6XHis-tagged protein coupled to Affigel-10 (Bio-Rad).

### Western Blotting

Each lane of a 10% SDS-PAGE gel was loaded with lysates from the following amount of tissues: five pairs of adult testes, or two ovary pairs, or five third instar larval brains. Samples were lysed in SDS-PAGE loading buffer, and heated at 100°C for 5 min, then centrifuged for 5 min to pellet insoluble debris. Proteins were separated using an SDS-PAGE minigel electrophoresis system (Bio-Rad) and transferred to UltraCruz 0.45 µm pore size nitrocellulose membrane (Santa Cruz) using Trans-Blot SD Semi-Dry Transfer system (Bio-Rad). The membrane was probed with rabbit anti-Sprn serum (1∶10,000 dilution) and mouse anti-α-tubulin DM1A antibody (Sigma, 1∶15,000) as a loading control. For secondary antibodies, IRDye800CW Goat anti-rabbit antibody and IRDye680LT Goat anti-mouse antibody (LI-COR Bioscience) were used. The membrane was scanned with an Odyssey Infrared Imaging System (LI-COR Bioscience).

### Immunostaining and microscopy

For immunostaining of testes, testes from adult males were dissected in Dulbecco's Phosphate Buffered Saline (DPBS, Invitrogen), then transferred to a 4 µl drop of DPBS on a slide and then covered with a siliconized coverslip containing 1 µl of 18.5% formaldehyde in DPBS. After allowing the tissue to flatten for 20–30 seconds under the weight of the coverslip, the slide was snap-frozen by plunging into liquid nitrogen. The slide was removed from liquid nitrogen and the coverslip was flipped off using a single-edged razor blade and then immersed immediately into −20°C methanol and incubated for 10 min. The slides were then transferred to PBS. A super pap pen (Immunotech) was used to draw a hydrophobic ring around the tissue. The tissues were stained with antibodies in 50 µl of PBS solution containing 5 mg/mL Bovine Serum Albumin (BSA) and 0.1% Saponin (Sigma). Kc167 cells were prepared for indirect immunostaining according to Kao and Megraw (2004) [16]. For immunostaining of mature sperm, seminal vesicles were dissected intact in DPBS and were then carefully peeled to release sperm in a 4 µl drop of DPBS on clean slides, the tissues were then treated as described in the immunostaining of testes. Samples were incubated with the following primary antibodies: affinity-purified rabbit anti-Sprn antibody (1∶1000), anti-ATP synthase complex V subunit alpha antibody (Abcam MS507, 1∶1000), mouse anti-γ-tubulin clone GTU88 (Sigma, 1∶1000), rabbit anti-Myc TRITC-conjugated antibody (Santa Cruz sc-789, 1∶100). Secondary goat antibody conjugates to Alexa 488,568, and 647 (Invitrogen, 1∶1000) were used. DNA was stained with DAPI (Invitrogen, 1 µg/ml). Samples were imaged using a Nikon A1 confocal microscope (Nikon, Japan) with a 60X/NA1.49 oil immersion objective. For superresolution imaging, DeltaVision OMX Blaze (Applied Precision, GE Healthcare) was used with an Olympus 60X/NA 1.42 objective.

### Cell culture

*Drosophila* Kc167 cells [17] were maintained in Hyclone CCM3 medium (Thermo Scientific) supplemented with 5% fetal bovine serum (Omega Scientific) and Penicillin-Streptomycin (Cellgro, 10,000 IU/mL Penicillin, 10,000 ug/mL Streptomycin). Cells were transfected with p*Actin5C-sprn-Myc* using Lipofectamine 2000 (Invitrogen) and prepared for immunostaining the next day after transfection.

### Male fertility test

Virgin *w^1118^* females and newly eclosed (less than eight hours old) males were collected and held apart for 3–5 days prior to mating. In each test, a single male was mated with a single *w^1118^* virgin female for 4 days. After 10 days, the progeny from each pair mating were counted. For each genotype, 3 groups, about 20 males from each were assessed. The whole test was conducted at 25°C. Error was measured as standard error of the means (SEM) and Student's t test was used to measure significance.

### Sperm tail measurements

*Drosophila* seminal vesicles were dissected intact in Shields and Sang M3 Insect Medium (Sigma) with 1 µg/ml Hoechst 33342 dye (Sigma) and were then carefully peeled to release sperm on clean slides. Single sperm were imaged with a Nikon Eclipse TE2000-U inverted microscope with a Plan Fluor 10X NA 0.30 phase contrast objective. The lengths of tails were then measured using Nikon Elements software. Approximately 10 sperm were measured from each pair of testes, and at least 3 pairs of testes were assessed for each genotype. Error was measured as standard error of the mean (SEM) and Student's t test was used to measure significance.

## Results

### Sprn is a testis-specific protein in Drosophila

*Spermitin* (*sprn*; also known as *CG14128*) expresses four mRNA splice variants predicted to encode 4 structurally similar protein variants (Sprn-PC, Sprn-PE, Sprn-PB and Sprn-PF) ranging from 544 to 579 amino acids with a predicted molecular weight of 61.1 kD to 65.1 kD (FlyBase.org) [18]. Conserved domain queries of Sprn revealed a Pleckstrin homology-like (PH) domain related to Ran binding domain in its C-terminus (Fig. 1A). We determined the transcription profile of *sprn* by RT-PCR from different organ extracts, which showed that *sprn* is transcribed in adult testes but not in ovaries or third instar larval brains (Fig. 1B). High-throughput expression data from the modENCODE and FlyAtlas Anatomical Expression Data on FlyBase.org are consistent with Sprn expression limited to males and restricted to the testis [18]–[22]. The RT-PCR of sequences at *sprn* showed three bands, two of which can be accounted for as transcripts RB/RC and RE/RF, and one that is not accounted for by the current gene model in Flybase. This suggests that there is an additional alternative splice product for *sprn* (Fig. 1A, B).

To characterize Sprn protein, we chose Sprn-PB as a representative because a cDNA clone was available. Moreover, there are very small differences among the 4 Sprn variants. We generated a rabbit polyclonal antibody against the N-terminal 200 amino acids of Sprn-PB, which is predicted to recognize all four Sprn variants (Fig. 1A). Western blotting showed that the antibody specifically recognizes a single protein band of approximately 68 kD in wild-type testes, and this band is consistent in size with the predicted Sprn products, which range between 61.1 kD to 65.1 kD. No Sprn band was detected in testes of a homozygous *sprn* mutant, *sprn^MB12149^*, containing a *minos* transposon insertion mutation that is mapped within the coding sequence in exon 2 of the *Sprn-RB* transcript and conceptually should block all protein products (Fig. 1A). No Sprn protein was detected in extracts from ovaries or larval brains (Fig. 1C), consistent with the absence of detectable transcripts outside of the testis [18]–[22]. The *minos* insertion mutation is predicted to produce a truncated Sprn protein that conceptually could be detected by our antibody (Fig. 1A), but no smaller protein was detected on blots from *sprn^MB12149^* mutant testes.

### Sprn is expressed in the nebenkern throughout spermatid elongation

**Figure 2:**
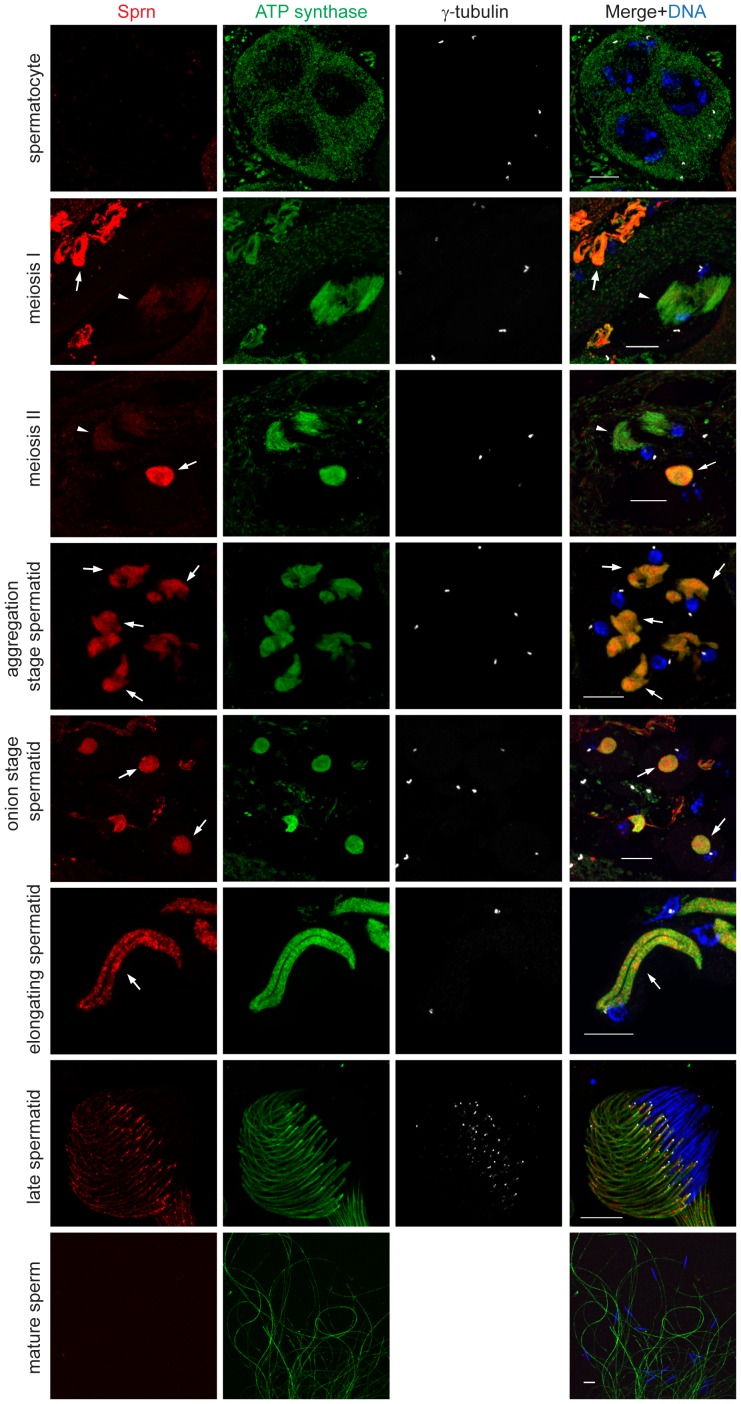
Sprn is expressed in the nebenkern throughout spermatid elongation. Endogenous Sprn expression is first detectable, though weak, in meiosis I and II, when mitochondria form first aggregates (arrowheads). Sprn expression increases at the spermatid nebenkern and persists throughout spermatid elongation (arrows), but is absent in mature sperm. ATP-synthase labels inner membranes of mitochondria. γ-tubulin labels the centriole as follows: in each spermatocyte, there are two v-shaped centriole pairs (four centrioles); in meiosis I, each daughter cell has one pair of centrioles (two centrioles); in meiosis II, each daughter cell has one centriole and after meiosis, each spermatid has only one centriole. Scale bars, 10 µm.

**Figure 3:**
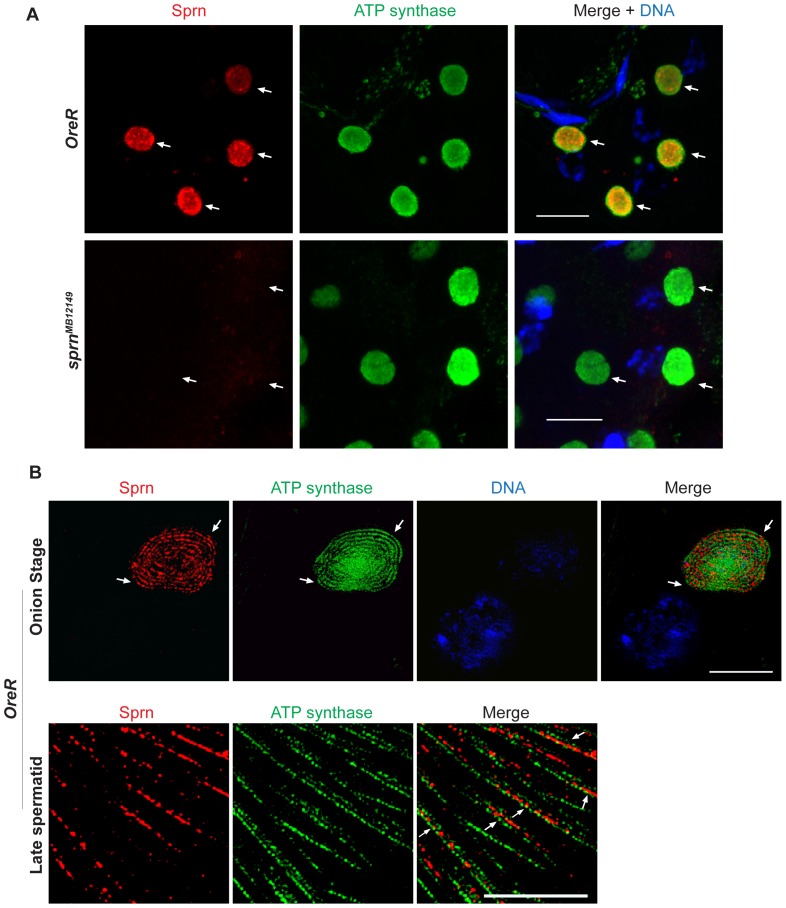
Sprn localizes in the nebenkern lumen. (A) Sprn is absent from *sprn^MB12149^* mutant nebenkerns (arrows). Scale bars, 10 µm. (B) Superresolution images showing Sprn localizes in the matrix of the nebenkern in spermatids. In the onion stage (upper panel), when two big mitochondrial derivatives wrap around one another to form the nebenkern, Sprn does not overlap with the mitochondrial inner membrane marker ATP synthase complex V subunit alpha (arrows). And in late spermatids (lower panel), when the mitochondrial derivative becomes a slender and cylindrical tube, Sprn is in the matrix of mitochondria, as shown localizing in-between the inner membranes marked by ATP synthase complex V subunit alpha in the longitudinal section (arrows). Scale bars, 5 µm.

To investigate the subcellular localization of Sprn, we used indirect immunofluorescent staining with affinity-purified Sprn antibodies on testes *in situ*. In wild-type (*w^1118^*) adult testes Sprn co-localized with the mitochondrial protein ATP-synthase subunit alpha at the nebenkern in spermatids. By counterstaining with the centriolar marker γ-tubulin to identify different developmental stages of the testis, we found that endogenous Sprn is expressed late in spermatocyte development, and appears by the onset of meiosis, where it localizes to mitochondria prior to nebenkern formation in spermatids. Sprn signal at the nebenkern in spermatids is conspicuously higher compared to late spermatocytes, and expression at the nebenkern persists throughout spermatid elongation (Fig. 2). This signal is specific because the homozygous *sprn^MB12149^* mutant has no detectable Sprn expression in spermatid mitochondria (Fig. 3A). We did not detect Sprn in mature sperm (Fig. 2), consistent with the previous finding that Sprn is not a component of the mature sperm proteome [23].

Superresolution structured illumination microscopy (SIM) imaging revealed a more detailed localization of Sprn at the nebenkern (Fig. 3B). SIM revealed that Sprn does not overlap with ATP synthase complex V subunit alpha at the mitochondrial inner membrane. Instead, Sprn appears to reside in the mitochondrial matrix (Fig. 3B).

### Ectopic expression of Sprn localizes at mitochondria

**Figure 4:**
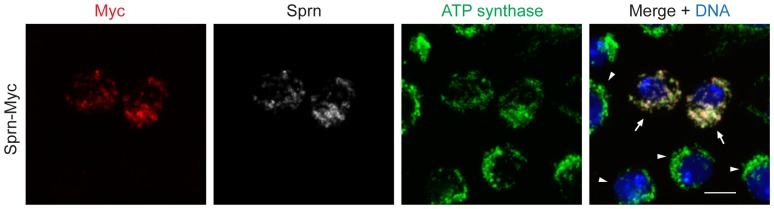
Ectopically expressed Sprn localizes to mitochondria. Sprn-Myc, expressed in Kc167 cells, colocalizes with mitochondrial marker ATP synthase (arrows). Sprn or Myc signal was absent from untransfected cells (arrowheads). Scale bars, 10 µm.

We sought to determine whether the mitochondrial localization of Sprn is conserved in different cell types. In transfected *Drosophila* Kc167 cells, Sprn-Myc co-localizes with ATP synthase complex V subunit alpha, confirming its localization at mitochondria (Fig. 4). Ectopic expression of Sprn in throughout the nervous system driven by elav-GAL4 produced no overt phenotype and flies developed into apparently healthy fertile adults. However, ubiquitous Sprn expression driven by tubulin-GAL4, actin5C-GAL4 or Ubiquitin-GAL4 was lethal (Table 1). The early lethality with the tubulin-GAL4 driver (prior to the third instar larval stage) relative to the ubiquitin driver (late pupae) likely reflects the different expression levels from the two drivers.

**Table 1:** Lethal analysis of ectopic *in vivo* UAS-Sprn-GFP expression.

Expression pattern	Parental crosses (virgin female×male)	F1	Assessment
Throughout nervous system	elav-GAL4×UAS-Sprn-GFP/TM6B	Out of 169 F1 flies, 92 were elav-GAL4/+; UAS-Sprn-GFP/+ or elav-GAL4/Y; UAS-Sprn-GFP/+, and they were apparently healthy and fertile.	No overt effect when expressed throughout the nervous system
Ubiquitous	tubP-GAL4/TM6B×UAS-Sprn-GFP/TM6B	Out of 97 F1 third instar larvae or pupae, no tubP-GAL4/Sprn-GFP. All progeny were Tb^1^ (TM6B).	Lethal prior to third instar larval stage
	UAS-Sprn-GFP/TM6B×tubP-GAL4/TM6B	Out of 196 F1 third instar larvae or pupae, no tubP-GAL4/Sprn-GFP. All progeny were Tb^1^ (TM6B).	Lethal prior to third instar larval stage
	Act5C-GAL4/SM5×UAS-Sprn-GFP/TM6B	Out of 106 F1 flies, no Act5C-GAL4/+; UAS-Sprn-GFP/+ progeny were produced. All progeny were Cy (SM5), Hu^1^ (TM6B), or both.	Lethal prior to eclosion (stage not determined)
	UAS-Sprn-GFP/TM6B×Act5C-GAL4/SM5	Out of 179 F1 flies, no Act5C-GAL4/+; UAS-Sprn-GFP/+ progeny were produced. All progeny were Cy (SM5), Hu^1^ (TM6B), or both.	Lethal prior to eclosion (stage not determined)
	Ubiquitin-GAL4×UAS-Sprn-GFP/TM6B	Out of 82 F1 pupae, 10 were Ubiquitin-GAL4/+; UAS-Sprn-GFP/+, and they all died before eclosion. The remaining pupae were Tb^1^ (TM6B).	Late pupal lethal
	UAS-Sprn-GFP/TM6B×Ubiquitin-GAL4	Out of 151 F1 pupae, 23 were Ubiquitin-GAL4/+; UAS-Sprn-GFP/+, nd they all died before eclosion. The remaining pupae were Tb^1^ (TM6B).	Late pupal lethal

All crosses were incubated at 25°C. The *UAS-Sprn-GFP* transgene is balanced over TM6B, Tb^1^ Hu^1^.

### Sprn is not essential for male fertility or sperm tail elongation

Previous studies have shown that particular mitochondrial proteins that are necessary for mitochondrial morphology or mitochondrial metabolism lead to defects in spermatogenesis and hence fertility in *Drosophila* [3], [6]–[11].

**Figure 5:**
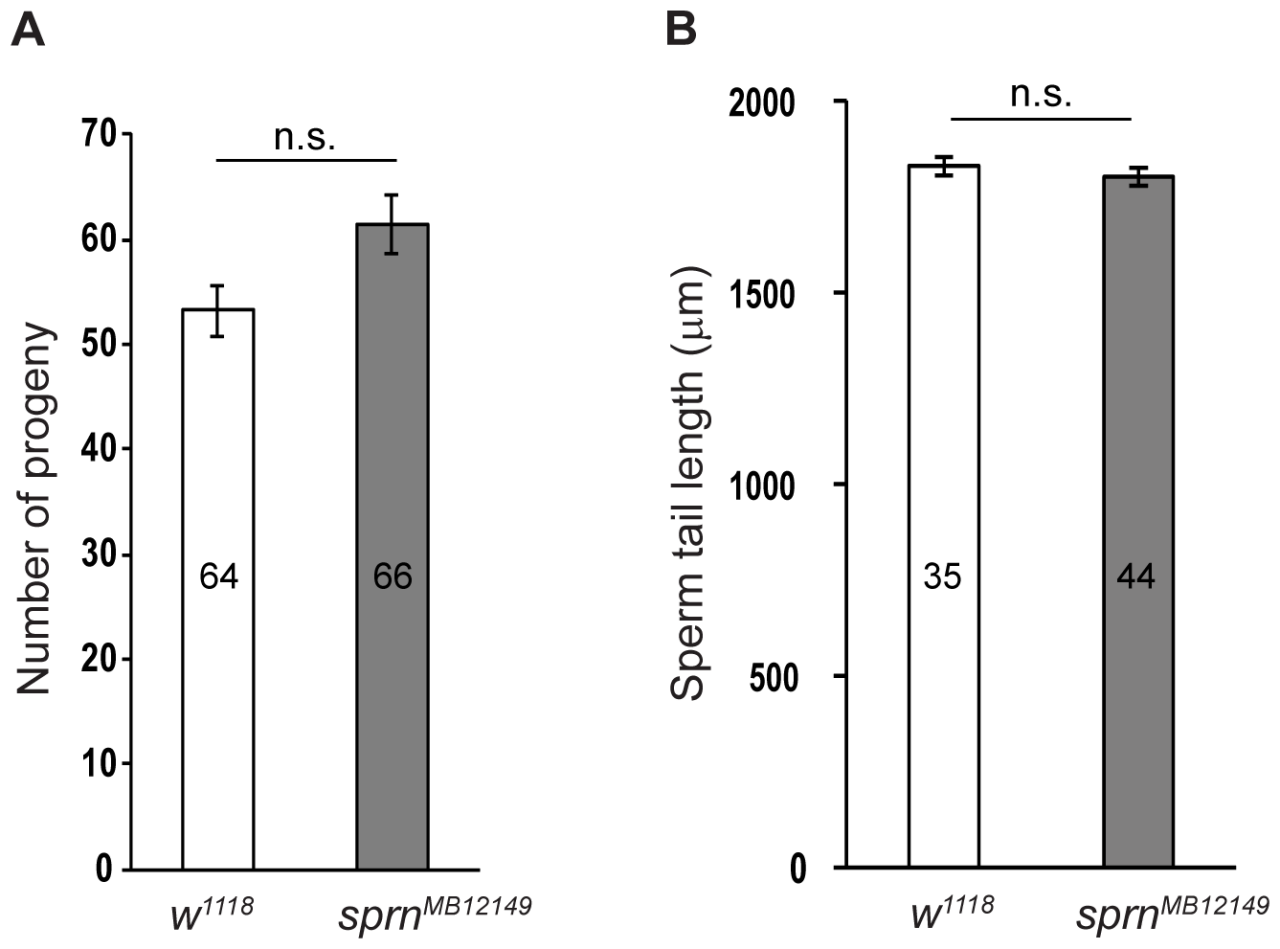
*Sprn* mutant males have normal fertility and sperm tail length. (A) The number of progeny produced by single *w^1118^* or *sprn^MB12149^* males in the fertility test. The *sprn^MB12149^* mutant males produced more progeny, but the difference was not significant according to Student's t test (p = 0.0905). Error bars represent standard error of the means (SEM), numbers inside the bars indicate the total number of males tested. (B) *sprn^MB12149^* has normal sperm tail length compared to wild-type (*w^1118^*). Error bars represent standard error of the means (SEM), Student's t test was used to determine the statistical significance (p = 0.438). Values inside the bars indicate the total number of sperm that were measured.

To determine whether the *sprn^MB12149^* mutation affects male fertility, we examined homozygous *sprn^MB12149^* adult males and their sperm. The homozygous *sprn^MB12149^* mutant can be maintained as a stock, indicating that loss of Sprn does not abolish male fertility. We then sought to determine whether the fertility of homozygous *sprn^MB12149^* males is reduced compared to the wild-type. Fertility tests showed that *sprn^MB12149^* male fertility is comparable to wild-type males (Fig. 5A). Consistent with male fertility, dissected *sprn^MB12149^* testes show an abundance of motile sperm (not shown). Since functional mitochondria are essential for efficient sperm tail elongation [5], we investigated whether the *sprn^MB12149^* mutation effects sperm tail length. We measured the tail length of mature sperm from seminal vesicles, and found that *sprn^MB12149^* males produce mature sperm with normal tail length, indicating that spermatid elongation is normal in the *sprn^MB12149^* mutant (Fig. 5B). Therefore, Sprn is not overtly essential for male fertility or sperm tail elongation.

## Discussion

In this study we describe Sprn, a testis mitochondrion-specific protein that localizes to the nebenkern, a unique mitochondrial fusion structure that forms in the spermatids of *Drosophila* and other insects. Sprn has a conserved Pleckstrin homology-like (PH) domain related to Ran-binding protein 1 at its C-terminus. BLAST searches revealed that putative orthologs exist in Drosophilids and other dipteran insects, but we could not find clear homologs in the genomes of other organisms. Possibly, Sprn evolved specifically with insects that have the nebenkern or similarly fused mitochondrial derivatives.

While the testis-specific expression of Sprn suggests it has a role in spermatid-specific functions like sperm elongation, our study shows that Sprn is not essential for generating sperm with robust fertility or normal tail length. Despite the lack of an overt mutant phenotype, a role for Sprn in spermatid mitochondria function or sperm cell development is possible given its restricted expression there. Ubiquitous expression of Sprn was lethal, while expression throughout the nervous system was not, suggesting that Sprn performs a function that is deleterious when executed outside of spermatogenesis in undetermined non-neuronal tissue types.
